# Expression of Neuropeptide F Gene and Its Regulation of Feeding Behavior in the Pea Aphid, *Acyrthosiphon pisum*

**DOI:** 10.3389/fphys.2018.00087

**Published:** 2018-02-13

**Authors:** Xiao Li, Ming-Jing Qu, Yi Zhang, Jian-Wen Li, Tong-Xian Liu

**Affiliations:** ^1^State Key Laboratory of Crop Stress Biology for Arid Areas, and Key Laboratory of Integrated Pest Management on the Loess Plateau of Ministry of Agriculture, Northwest A&F University, Yangling, China; ^2^Shandong Peanut Research Institute, Qingdao, Shandong, China; ^3^College of Life Science, Yangtze University, Jingzhou, China

**Keywords:** neuropeptide F, neuropeptide F receptor, transcriptional expression, regulation of feeding, *Acyrthosiphon pisum*

## Abstract

Neuropeptide F (NPF) signaling systems are widespread and highly evolutionarily conserved from vertebrates to invertebrates. In fact, NPF has been identified in many insect species and plays regulatory roles in diverse physiological processes, such as feeding, learning, reproduction and stress responses. NPF operates by interacting with the NPF receptor (NPFR). Here, we characterized and determined the presumed role of NPF signaling in the wingless parthenogenetic pea aphid, *Acyrthosiphon pisum*. Quantitative real-time reverse transcription-PCR (qRT-PCR) revealed that the expression levels of both NPF and NPFR transcripts varied across developmental stages, which implies that the NPF signaling system might participate in the developmental regulation of aphid physiological processes or behaviors. The NPF transcript was mainly detected in the head but not in the gut, whereas the NPFR transcript was mainly detected in both the gut and head. In addition, the NPF transcript levels were markedly up-regulated in starved aphids compared with satiated aphids, and the transcript levels recovered after re-feeding. In contrast, the NPFR transcript levels remained stable in starved and re-fed aphids. Furthermore, RNAi knockdown by the injection of NPF dsRNA into wingless adult aphids significantly reduced their food intake. Further analysis of the modification of aphid feeding behavior on broad bean plants using electrical penetration graphs (EPGs) revealed that both the probing time and the total duration of phloem activity decreased significantly in the NPF treatment group. These results indicated a lower appetite for food after NPF knockdown, which could explain the reduction in aphid food intake. NPF silencing was also shown to reduce reproduction but not survival in aphids. Overall, the results of these experiments suggest that NPF plays an important role in regulation of feeding in *A. pisum*.

## Introduction

Neuropeptide Y (NPY) is an important multipeptide molecule that acts as a neurotransmitter/neuromodulator to regulate the physiology and behavior of vertebrates (Cerdá-Reverter and Larhammar, 2000). Neuropeptide F (NPF) is the ortholog of NPY in invertebrates and shows high similarity to NPY in terms of both structure and function, i.e., it has similar physiological functions as NPY and only differs in having a phenylalanine (F) at the C-terminus instead of a tyrosine (Y) (Maule et al., 1991; Rajpara et al., 1992; de Jong-Brink et al., 2001). NPF was initially found in a flatworm, *Moniezia expansa* (Maule et al., 1991). Through the use of a specific antiserum, insect NPF-related peptides were isolated from the Colorado potato beetle, *Leptinotarsa decemlineata* (Spittaels et al., 1996) and the American cockroach, *Periplaneta americana* (Veenstra and Lambrou, 1995). However, these short peptides of 8–10 amino acids in length were subsequently found to be encoded by another gene, the short NPF (sNPF), instead of the NPF-encodinig gene. The first real insect NPF (long NPF) was identified in *Drosophila melanogaster* based on a radioimmunoassay for a gut peptide of the corn earworm, *Helicoverpa zea* (Brown et al., 1999). Since then, NPF genes have been found in 39 insect species (Yeoh et al., 2017), including *Aedes aegypti* (Stanek et al., 2002), *Anopheles gambiae* (Garczynski et al., 2005), *Locusta migratoria* (Clynen et al., 2006), *Bombyx mori* (Roller et al., 2008), and *Reticulitermes flavipes* (Nuss et al., 2010).

NPFs are evolutionarily conserved. Most consist of 28–45 residues and are characterized by an RxRFamide sequence at the C-terminus (Nässel and Wegener, 2011) and exert their effects through interaction with NPF receptors, which are members of the G protein-coupled receptor superfamily. NPFs are known to play a very important regulatory role in feeding and foraging behaviors (Shen and Cai, 2001; Wu et al., 2003, 2005b). In fact, regulation of feeding was the first documented role of NPF, and most of the related information was obtained in studies with *D. melanogaster* (Brown et al., 1999; Wu et al., 2003, 2005a,b). For instance, the NPF signaling system regulates the acceptance of noxious food in *Drosophila*. Fly larvae that overexpress NPFR are more likely to consume noxious food, while loss of NPFR results in the opposite phenotype (Wu et al., 2005b). Similarly, the NPF signaling system is required for fly larvae to exhibit feeding behavior under harmful cold conditions (Lingo et al., 2007). This feeding-related function has also been documented in several other insect species (Stanek et al., 2002; Gonzalez and Orchard, 2008; Nuss et al., 2008, 2010; Huang et al., 2011; Van Wielendaele et al., 2013a). In addition, NPFs are involved in other functions, such as ethanol sensitivity (Wen et al., 2005), reproduction (Lee et al., 2006; Van Wielendaele et al., 2013b; Sedra and Lange, 2016), circadian rhythm (Lee et al., 2006; Hermann et al., 2012; Erion et al., 2016), aggression (Dierick and Greenspan, 2007), and learning and memory (Krashes et al., 2009).

Through immunocytochemistry, *in situ* hybridization and quantitative real-time reverse transcription PCR (qRT-PCR) experiments, NPF has been shown to be secreted mainly by the central nervous system (CNS) and the midgut (Brown et al., 1999; Stanek et al., 2002; Nuss et al., 2008; Van Wielendaele et al., 2013a). For instance, NPF occurs in relatively few brain cells and many midgut endocrine cells in *D. melanogaster* larvae and adults (Brown et al., 1999). However, there has been less research on the localization and distribution of NPFR. In *Drosophila* larvae, NPFR is mainly expressed in neurons in the brain and ventral nerve cord and in midgut cells, whereas it is expressed in the central and peripheral nervous systems in embryos and adults (Garczynski et al., 2002; Feng et al., 2003).

Although the NPF signaling system has been investigated in several insect species, information on the localization and functional roles of NPF in aphids is lacking. However, a comprehensive analysis of the pea aphid, *Acyrthosiphon pisum*, genome and the EST database revealed the presence of an NPF ortholog consisting of 45 amino acids with a typical RPRFamide C-terminus (Huybrechts et al., 2010).

In this study, the transcript levels of the NPF and NPFR genes were investigated following starvation and subsequent re-feeding to determine whether this signaling was affected by the feeding state of the pea aphid. To further examine the possible relationship between NPF signaling and the feeding behavior of the pea aphid, the NPF-transcript levels were knocked down via RNAi, and the results revealed that silencing of the *A. pisum* NPF gene inhibited food intake and caused changes in probing behavior and phloem ingestion on *Vicia faba* seedlings.

## Materials and methods

### Insect rearing

The strain of pea aphids (*A. pisum*) used in this study, which have a green body color, was obtained from a long-established parthenogenetic colony in our laboratory (originally collected from *V. faba* in New York, USA in 2009). The insects were maintained on 2–3-week-old faba bean plants in a climate cabinet (Jiangnan, Ningbo, China) under standardized conditions of 19 ± 1°C, 70% relative humidity (RH) and a 16-h light/8-h dark photoperiod, and were reared at a low density (~10 aphids per seedling) to avoid the generation of winged individuals. The aphid developmental stages were synchronized by collecting newborn aphid nymphs for 12 h after apterous adult aphids were placed on fresh broad bean leaves.

### Analysis of NPF and NPFR transcript levels during different life stages and in different body parts via qRT-PCR

To monitor the transcriptional expression levels of NPF and NPFR during different life stages, at least 20 aphids from each instar were collected separately. To measure the transcriptional levels of these two genes in selected body parts, ~30–40 synchronized wingless adult aphids were dissected in 0.1 M phosphate-buffered saline (PBS, pH 7.4) under a stereoscopic microscope (Olympus Corporation, Tokyo, Japan). The head, gut, embryo and remaining body parts were collected separately.

RNA was extracted using RNAiso Plus (Takara, Dalian, Liaoning, China) according to the manufacturer's instructions. Reverse transcription was performed using the PrimeScript® RT Reagent Kit with gDNA Eraser (Takara, Dalian, Liaoning, China) in a 20 μl reaction mixture containing 800 ng of total RNA.

Relative expression levels were assayed via qRT-PCR using gene-specific primers (Table 1) and SYBR Premix Ex Taq II (Tli RNaseH Plus) (Takara, Dalian, Liaoning, China) on an iQ™5 Multicolor Real-Time PCR Detection System (Bio-Rad Hercules, CA, USA). Appropriate primers were designed using Primer Premier 5 software (Premier Biosoft, Palo Alto, CA, USA) and purchased from Invitrogen. The amplified sequence was different from that used to produce dsRNA. The ribosomal protein L7 (RPL7)-encoding gene (NM_001135898.1; Nakabachi et al., 2005) was used as a reference gene, and the relative quantification of the transcript levels was calculated using the 2^−ΔΔ*Ct*^ method (Livak and Schmittgen, 2001). Four or five biological replicates were conducted, and each was analyzed in triplicate.

**Table 1:** PCR primers for qRT-PCR and dsRNA synthesis.

**Usage**	**Primer name**	**Forward primer (5′−3′)**	**Reverse primer (5′−3′)**
qRT-PCR	NPF	CCGTGACGTCTACTGAAGTG	CCTTGGACGAGAAACTACCG
qRT-PCR	NPFR	CGATACTGGGTGATCGTCCG	ATAAGCTACGGGCGATGTGG
qRT-PCR	RPL7	GCGCGCCGAGGCTTAT	CCGGATTTCTTTGCATTTCTTG
dsRNA synthesis	NPF	GGCGGTAGTTTCTCGTCCAA	ATTTCCGTTTTCGCGACGTG
dsRNA synthesis	NPFR	ACGGACCACCCTACTTCTGA	TCCTGACTCCCAGGCATGTA
dsRNA synthesis	LTA	CACCCTCTCCACGAATTG	TAGAAGATGCTGCTGTTTCA

### Analysis of NPF and NPFR transcript levels under starvation and subsequent re-feeding via qRT-PCR

The transcriptional expression levels of NPF and NPFR were investigated following starvation treatment for 24 and 48 h. Specifically, ~15 wingless adult aphids from each treatment were placed on the abaxial surface of broad bean leaves in clip-cages (three aphids per cage and one cage per leaf). Each clip-cage was 3.5 × 1.5 cm (d × h) in size and was covered with fine mesh at the top. For the starvation group, we placed three layers of fine mesh between the leaf and the aphids inside the cage to prevent the aphids from reaching the leaf to feed. Aphids fed *ad libitum* were used as the first control, and those reared in the clip-cages were used as the second control. The same measurement was performed for the aphids that were re-fed in leaf cages for 12 and 24 h after 48 h of starvation. The whole bodies of aphids from each treatment were collected for RNA extraction. Both RNA extraction and qRT-PCR were performed using the methods described in section Analysis of NPF and NPFR Transcript Levels during Different Life Stages and in 106 Different Body Parts via qRT-PCR.

### dsRNA synthesis and injection

A 232-bp dsRNA representing the *A. pisum* NPF-encoding gene sequence (XM-001944830) and a 354-bp dsRNA representing the *A. pisum* NPFR-encoding gene sequence (XM_008185310.2) were synthesized using the T7 RiboMAX™ Express RNAi System (Promega, Madison, WI, USA) according to the manufacturer's instructions. To prevent off-target effects, we chose these sequences to avoid any overlap with other *A. pisum* genes exceeding 19 bp. The primers used for synthesis were designed with Primer Premier 5 software (Premier Biosoft, Palo Alto, CA, USA) and are listed in Table 1. We employed dsRNA representing the *Mus musculus* lymphotoxin A (LTA)-encoding gene (Gene ID: 16992; Chen et al., 2016; Wang et al., 2016), which does not have a natural homolog in *A. pisum*, as a control.

Wingless adult aphids on the 3rd day after reaching the adult instar stage were chosen for the RNAi microinjection assay. We injected 101.2 nl of dsRNA (~6 μg/μl) solution under a stereomicroscope using a Nanoject II micro-injector (Drummond Scientific Co., Broomall, PA, USA), and the glass capillary tubes (3.5-inch 3-000-203-G/X micropipettes, Drummond Scientific Co., Broomall, PA, USA) used for injection were pulled with a Flaming/Brown P-97 micropipette puller (Sutter Instrument Co., Novato, CA, USA). Prior to injection, the aphids were immobilized on a homemade adhesive tape by their dorsal thorax. The injection site was the lateral abdomen between the 2nd and 3rd abdominal segments at leg height (Sapountzis et al., 2014).

### Determining NPF and NPFR expression levels after dsRNA injection

After dsRNA injection, the aphids were returned to the broad bean plants on which they were reared, and RNA was isolated from the whole bodies of the aphids at 1, 2, 4, and 6 days after injection. For each treatment, 20 aphids were collected and immediately frozen in liquid nitrogen, and the NPF and NPFR mRNA levels were measured via qRT-PCR as described above.

### Honeydew excretion assay

Because the aphid weight will not show obvious variation due to hunger over a short time during the adult stage, we selected the amount of honeydew excretion as the parameter for measuring changes in aphid food intake (Lei et al., 2014). Therefore, the difference in honeydew excretion between the aphids injected with NPF dsRNA and those injected with LTA dsRNA was examined to determine the influence of changes in the NPF expression levels on aphid feeding. Seventy-two hours after injection, 30 injected adult aphids from each group were transferred to leaf discs (3.5 cm in diameter), which were made as described by Will and Vilcinskas (2015), with two individuals per disc, and non-injected aphids were also chosen as a control. A pre-weighed piece of aluminum foil was placed in the Petri dish covers to collect the honeydew excreted by the aphids. The leaf discs were maintained in a climate cabinet under the conditions described above, and the leaves were replaced every 3 days. After honeydew had been collected for 24 h (from the 3rd to 4th day after dsRNA injection), the pieces of aluminum foil were placed in a drying oven at 50°C for 4 h and reweighed using an analytical balance with microgram sensitivity (Sartorius, Göttingen, Germany).

### EPG analysis of aphid feeding behavior

The feeding behavior of aphids in the NPF treatment group and LTA control group was compared using the electrical penetration graph (EPG) technique (McLean and Kinsey, 1964; Tjallingii, 1978). Two to four days after injection, a gold wire electrode (2 cm × 18 μm) was attached to the dorsum of randomly selected aphids using electrically conductive silver glue, and the electrodes were connected to a Giga-8 DC EPG system (Tjallingii, 1978). The EPG output was recorded with Stylet+ (hardware and software from EPG Systems, Wageningen, Netherlands). The plant electrode was inserted into the soil of a potted plant. The entire experimental setup was placed in a Faraday cage to shield it from electromagnetic interference. Aphids were placed on the backside of a mature leaf on a 2-week-old faba bean, and EPG recordings were immediately started and run for 8 h. After the EPG assay, the aphids were immediately frozen in liquid nitrogen to measure the NPF mRNA level via qRT-PCR. The data from the aphids that showed no effective down-regulation of the NPF mRNA level were excluded from the analysis, and EPG waveforms were analyzed using the Stylet+ analysis module as previously described (Prado and Tjallingii, 1994). Further analysis was performed using Excel Workbook for automatic parameter calculation of EPG data 4.3 (Sarria et al., 2009).

### Survival and reproduction assay

Survival and reproduction assays were conducted simultaneously using 30 aphids per treatment group. The aphids were reared in leaf discs as described above, and the numbers of adult deaths and of newborn nymphs per adult aphid were recorded once per day beginning on the first day after dsRNA injection. The leaf discs were placed in a climate cabinet under the conditions described above.

### Statistical analysis

One-way analysis of variance (ANOVA) was employed to compare the qRT-PCR data obtained from the expression pattern assays of the NPF and NPFR genes, and post-hoc comparisons of the means were performed using Duncan's test. Additionally, ANOVA followed by Duncan's test was used to compare the data on the changes in the amount of honeydew. Student's *t*-test was employed to compare the data on the changes in the NPF and NPFR expression levels after dsRNA injection. The survival data were subjected to a Kaplan-Meier survival log-rank analysis, and the reproduction data were analyzed via ANOVA. The non-parametric Mann-Whitney test was used to analyze the feeding behavior data from the EPG recording, and the significance level for the statistical tests was set to *P* = 0.05. SPSS 20.0 (Systat Software Inc., London, UK) was employed for the statistical analyses, and SigmaPlot 11 (Systat Software Inc., London, UK) was used to construct the histograms.

## Results

### Transcriptional expression levels of NPF and NPFR during different life stages and in different body parts of pea aphids

**Figure 1:**
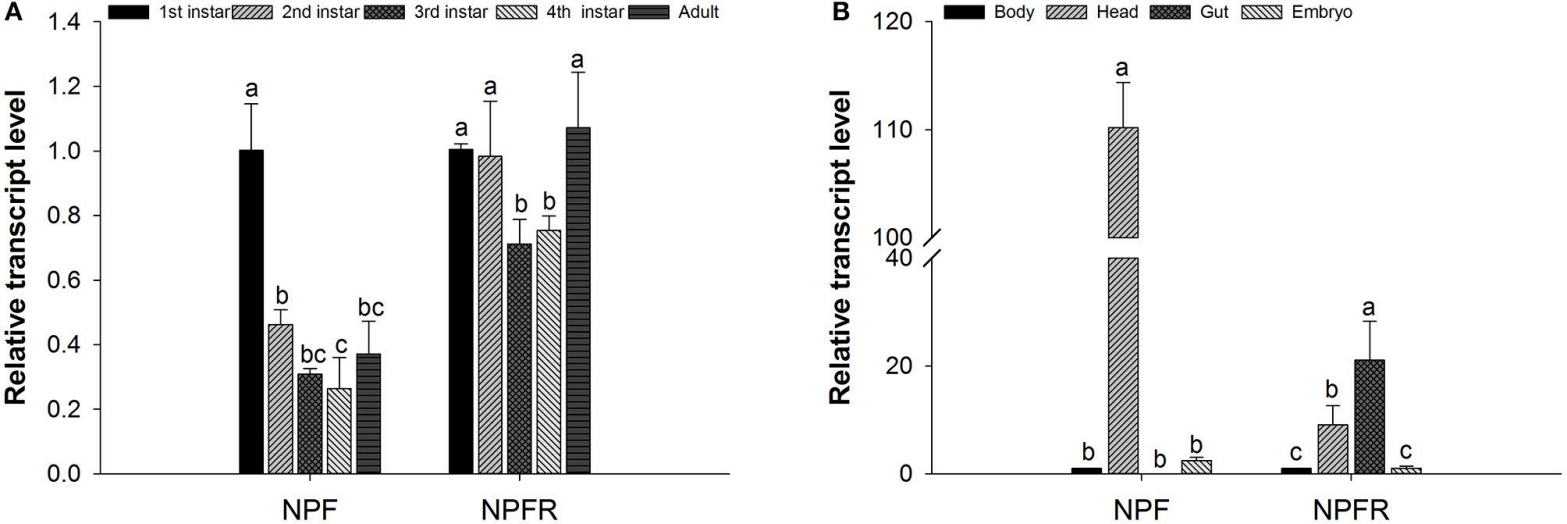
Relative expression levels of the NPF and NPFR transcripts at different developmental stages **(A)** and in different body parts **(B)** of *Acyrthosiphon pisum*. For each sample, the transcript level was measured via qRT-PCR and normalized against RPL7. The data were statistically analyzed by one-way ANOVA followed by Duncan's multiple-range test. Different lowercase letters indicate significant differences at the 0.05 level.

Before examining the effects of RNA interference on the NPF and NPFR genes in pea aphids, qRT-PCR experiments were performed to investigate the transcript profiles of these two genes. The expression levels of the two target genes normalized to that of the reference gene RPL7 are shown in Figure 1. The NPF transcript was detected at the highest expression level in 1st instar nymphs, with the statistical analysis indicating a significant difference (*F* = 31.815, df = 4, 10, *P* < 0.05). The NPFR transcript showed similar expression levels in the 1st and 2nd instar nymphs and adult aphids; these levels were significantly higher than the levels in the 3rd and 4th instar nymphs (*F* = 5.897, df = 4, 10, *P* < 0.05).

The expression levels of both the NPF and NPFR transcripts showed significant variation among the samples of the different body parts (head, gut, embryo and the remaining body) dissected from the pea aphids (Figure 1B). The highest NPF transcript level was observed in the head, whereas the NPF transcript was almost undetectable in the gut (*F* = 1318.362, df = 3, 8, *P* < 0.05). In contrast, the NPFR transcript was mainly expressed in the aphid intestine and head, and its transcription level in the intestine was approximately twice that in the head (*F* = 11.063, df = 3, 8, *P* < 0.05).

### Variation in the NPF and NPFR transcript levels in starvation and re-feeding assays

**Figure 2:**
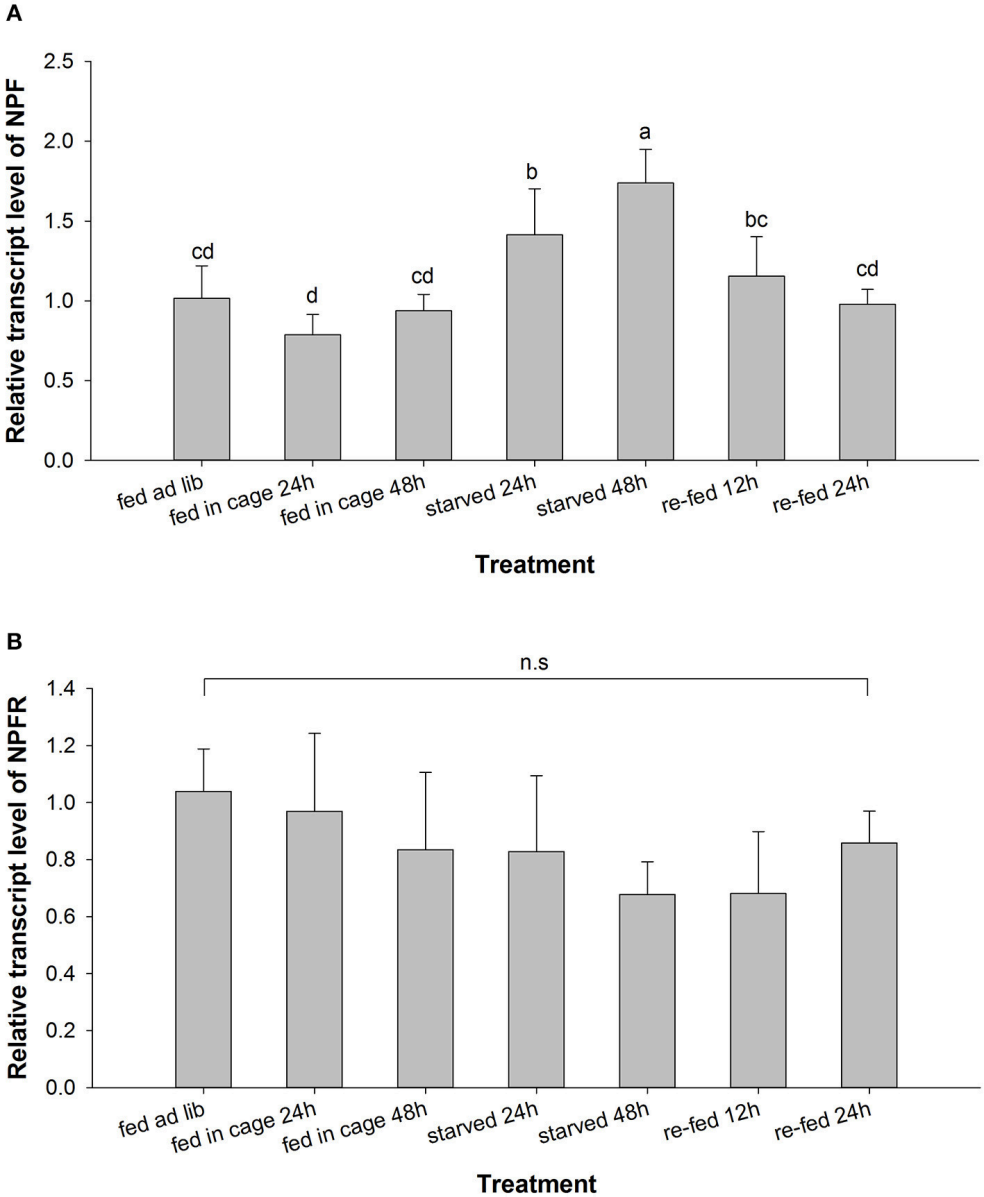
Relative expression levels of the NPF **(A)** and NPFR **(B)** transcripts in *Acyrthosiphon pisum* after starvation and subsequent re-feeding treatment. For each sample, the transcript level was measured via qRT-PCR and normalized against RPL7. The data were statistically analyzed by one-way ANOVA followed by Duncan's multiple-range test. Different lowercase letters indicate significant differences at the 0.05 level; n. s., not significant.

Because NPF signaling has been shown to be closely related to the feeding and nutritional status of the diverse insect species studied thus far, we established several time points for detecting the variation in the expression level of the NPF and NPFR genes in pea aphids after starvation and subsequent re-feeding experiments. Neither NPF nor NPFR showed a significant difference in transcriptional expression between the two control groups (fed in leaf cages and fed *ad libitum*). The expression levels of these two genes in wingless adult aphids responded differently to starvation stress (Figure 2). The NPF transcript level was significantly up-regulated in the starved groups compared with the fed groups and was higher at 48 h than at 24 h. Furthermore, during the re-feeding experiment following food deprivation, the NPF transcript level gradually decreased and then returned to its original level after 24 h of re-feeding (*F* = 9.516, df = 6, 18, *P* < 0.05, Figure 2A). However, the mRNA level of the NPF receptor gene showed no significant change in either the starvation experiment or the subsequent re-feeding experiment (*F* = 1.209, df = 4, 16, *P* > 0.05, Figure 2B).

### Variation in the NPF and NPFR transcript levels after dsRNA injection

**Figure 3:**
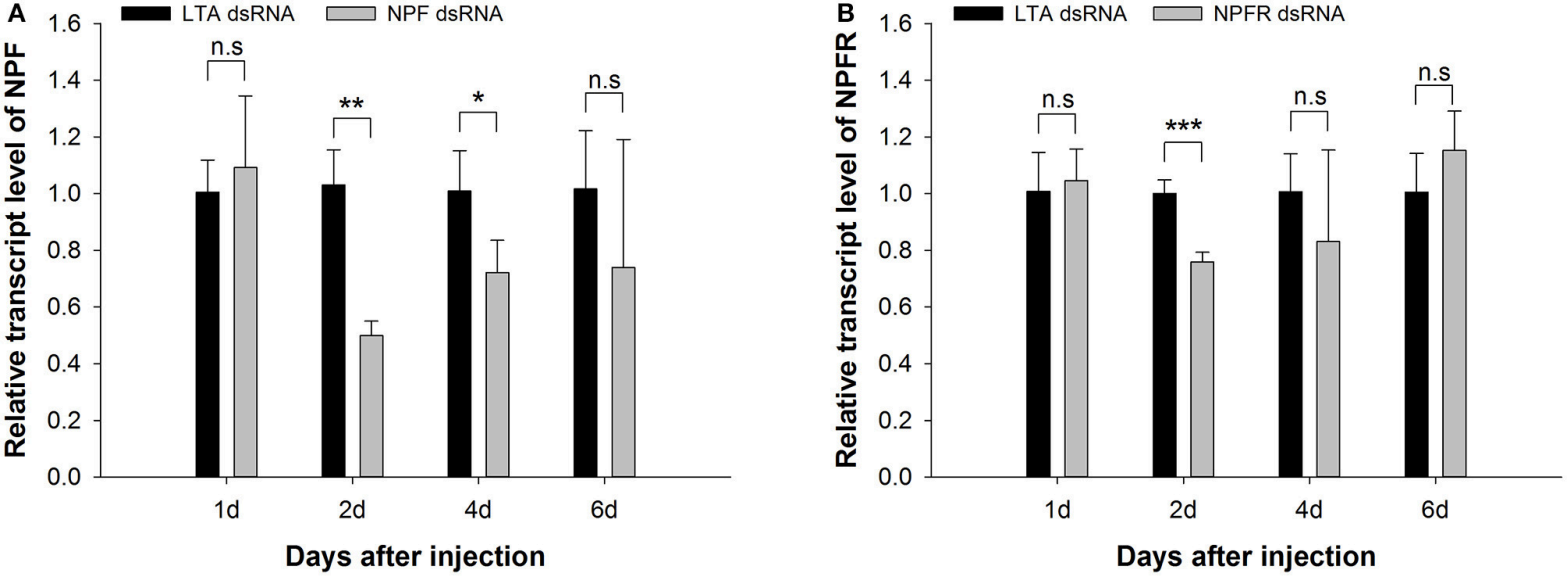
Knockdown of NPF **(A)** and NPFR **(B)** expression levels in *Acyrthosiphon pisum*. The transcript levels in pea aphids injected with NPF (or NPFR) dsRNA and LTA dsRNA (control) were measured via qRT-PCR and normalized against RPL7. The data were statistically analyzed by Student's *t*-tests: n. s., not significant; ^*^P < 0.05; ^**^P < 0.01; ^***^P < 0.001.

We measured the changes in the expression of NPF and NPFR at the mRNA level through qRT-PCR after the administration of dsRNA via microinjection. The data were analyzed for each of the time points described above (Figure 3). A significant reduction in the NPF expression level of ~50% was observed 2 days after dsRNA injection (*t* = 6.865, df = 6, *P* < 0.01, Figure 3A), and the inhibitory effect of NPF dsRNA was still detected on the 4th day after microinjection (*t* = 3.149, df = 6, *P* < 0.05). However, the effect of the knockdown of NPFR dsRNA through the microinjection method was not strong or long-lasting compared with that of NPF dsRNA, and the reduction of the expression level was only detected at the 2-day time point, with almost 20% inhibition (*t* = 8.231, df = 6, *P* < 0.001, Figure 3B).

### Influence of NPF silencing on pea aphid honeydew excretion

**Figure 4:**
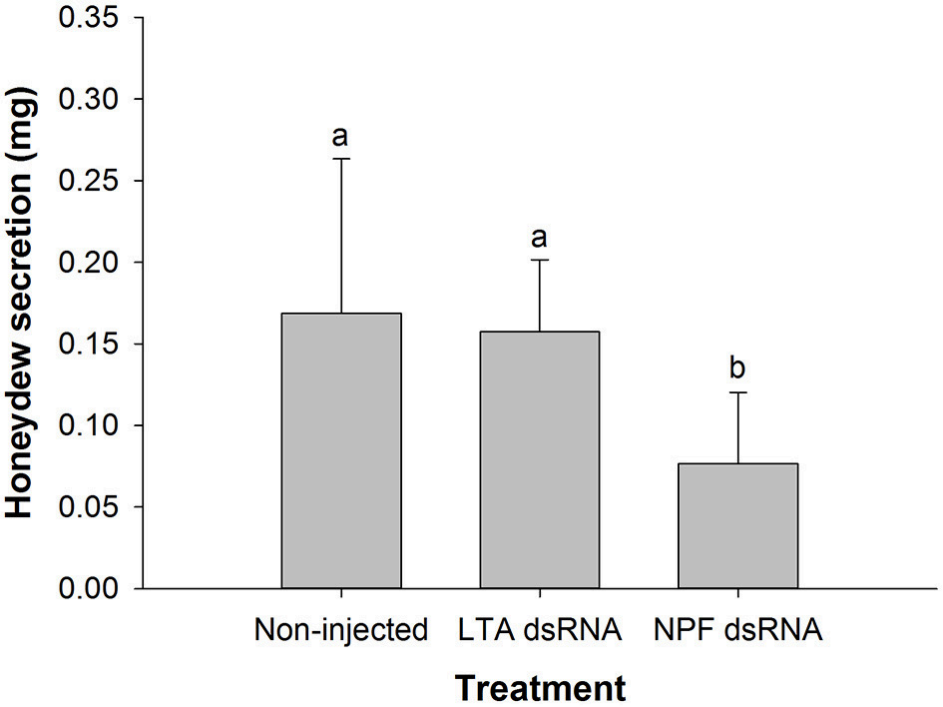
Influence of NPF gene silencing on honeydew excretion by *Acyrthosiphon pisum*. Honeydew was collected for 24 h (from the 3^rd^−4th day after injection). The data were statistically analyzed by one-way ANOVA followed by Duncan's multiple-range test. Different lowercase letters indicate significant differences at the 0.05 level.

We selected the NPF gene over its receptor gene because the former was more obviously down-regulated, facilitating the observation of phenotypic changes. There was no significant difference in the amount of honeydew collected per adult within 24 h between the LTA group (0.157 ± 0.044 mg) and the non-injected group (0.169 ± 0.095 mg, *P* > 0.05). However, the NPF treatment group (0.077 ± 0.044 mg) exhibited a significant reduction compared with the two control groups (*F* = 7.363, df = 2, 33, *P* < 0.01, Figure 4). This result indicated that the RNAi-mediated knockdown of NPF had an inhibitory effect on aphid food intake.

### Influence of NPF silencing on pea aphid feeding behavior

The changes in the probing and feeding behavior of pea aphids under NPF silencing treatment, as indicated by 33 EPG parameters, are shown in Table 2. Regarding stylet activity before reaching the phloem, the initiation of penetration was postponed on average from ~ 8 min in the LTA control group to ~84 min in the NPF group, as indicated by the “Time from start of EPG to 1st probe” (*P* = 0.006). However, the number of stylet probes before reaching phloem did not differ between the two groups, as indicated by the “Number of probes to the 1st E1” (*P* = 0. 173). Regarding the phloem-related parameters, the first activity in the phloem was postponed on average from ~ 3.3 to 5.6 h, as indicated by the “Time from start of EPG to 1st E” (*P* = 0.030). The total durations of the E and E2 waveforms were clearly reduced (*P* = 0.029 and *P* = 0.029, respectively), and correspondingly, the total duration of the no phloematic phase significantly increased (*P* = 0.029). However, the numbers of E1 or E2 waveforms did not differ (*P* = 0.059 and *P* = 0.091, respectively), and no differences in the mean duration of the E1 or E2 waveform were observed between the groups (*P* = 0.918 and *P* = 0.349, respectively). Overall, there was no difference in the number of probes between the NPF group and the controls (*P* = 0.488), but the total probing time was significantly decreased in the NPF group (*P* = 0.028).

**Table 2:** Comparison of probing and feeding behaviors of *Acyrthosiphon pisum* in the NPF treatment and LTA control groups on broad bean seedlings based on EPG recordings.

** Tissue specificity**	**Parameters**	**LTA**			**NPF**			***P*-value**
		**N**	**Mean [s]**	**SE [s]**	**N**	**Mean [s]**	**SE [s]**	
**(*FROM INITIATION OF EPG TO REACHING PHLOEM*)**									
Epidermis	Time from start of EPG to 1st probe	12	471.06	138.68	16	5017.38	2347.68	0.006^*^
Epidermis and mesophyll	Time from 1st probe to 1st E	14	11303.72	2802.33	18	15694.91	2452.20	0.488
All tissues	Time from 1st probe to 1st sustained E2 (>10 min)	14	13065.92	2861.73	18	15822.80	2443.62	0.750
Epidermis and mesophyll	Duration of 1st probe	14	2406.10	611.06	16	1542.58	988.46	0.193
Epidermis and mesophyll	Duration of non-probe period before the 1st E	14	3931.79	1413.14	18	8967.41	2024.54	0.220
Epidermis and mesophyll	Number of probes to the 1st E1	11	7.37	2.15	10	9.80	1.71	0.173
All tissues	Number of probes	14	12.21	2.54	18	15.11	2.71	0.488
All tissues	Total probing time	14	24388.43	1434.09	16	18077.85	2020.70	0.028^*^
All tissues	Number of short probes (C <3 min)	14	6.00	1.70	18	6.39	1.74	0.955
All tissues	Number of C	14	15.36	2.44	18	16.50	2.73	0.750
All tissues	Total duration of C	14	8481.21	941.69	16	9034.27	1193.74	0.580
All tissues	Mean duration of C	14	657.79	79.81	16	572.87	94.53	0.313
All tissues	Number of np	14	12.57	2.62	18	15.61	2.69	0.442
All tissues	Total duration of np	14	4411.57	1434.09	18	9530.80	1967.35	0.145
All tissues	Mean duration of np	14	353.83	69.00	16	736.69	187.99	0.120
All tissues	Total duration of no phloem phase	11	16039.60	1698.79	10	21669.38	1439.14	0.029^*^
Epidermis and mesophyll	Number of F	14	1.50	0.34	18	0.61	0.18	0.041^*^
Epidermis and mesophyll	Total duration of F	11	7485.14	1415.51	8	9173.89	1680.23	0.351
Epidermis and mesophyll	Mean duration of F	11	4014.00	507.47	8	7750.93	1977.33	0.129
**(*RELATED TO PHLOEM ACTIVITY*)**									
Epidermis and mesophyll	Time from start of EPG to 1st E	14	11707.48	2728.32	18	20154.81	2256.02	0.030^*^
All tissues	Time from start of EPG to 1st E2	14	12785.54	2742.85	18	20208.83	2249.56	0.065
All tissues	Time from start of EPG to 1st sustained E2 (>10 min)	14	13346.27	2792.28	18	20208.83	2249.56	0.099
Phloem	Number of E1	14	2.50	0.60	18	1.11	0.30	0.059
Phloem	Number of E2	14	2.21	0.52	18	1.11	0.30	0.091
Phloem	Number of single E1	14	0.29	0.13	18	0.00	0.00	0.180
Phloem	Number of sustained E2 (>10 min)	14	2.00	0.51	18	0.83	0.22	0.054
Phloem	Total duration of E	11	12760.41	1698.79	10	7130.62	1439.14	0.029^*^
Phloem	Total duration of E1	11	293.03	58.00	10	187.79	26.48	0.223
Phloem	Total duration of E2	11	12467.38	1703.66	10	6942.83	1441.79	0.029^*^
Phloem	Mean duration of E1	11	91.77	6.17	10	104.75	16.74	0.918
Phloem	Mean duration of E2	11	6327.05	1934.88	10	3882.93	934.09	0.349
Phloem	Duration of 1st E	11	4181.95	2118.90	10	5629.70	1219.59	0.085
Phloem	Duration of the longest E2	11	8878.54	1882.41	10	5532.46	1225.23	0.132

*The data were statistically analyzed using the non-parametric Mann-Whitney test*:

* *P < 0.05*.

**Figure 5:**
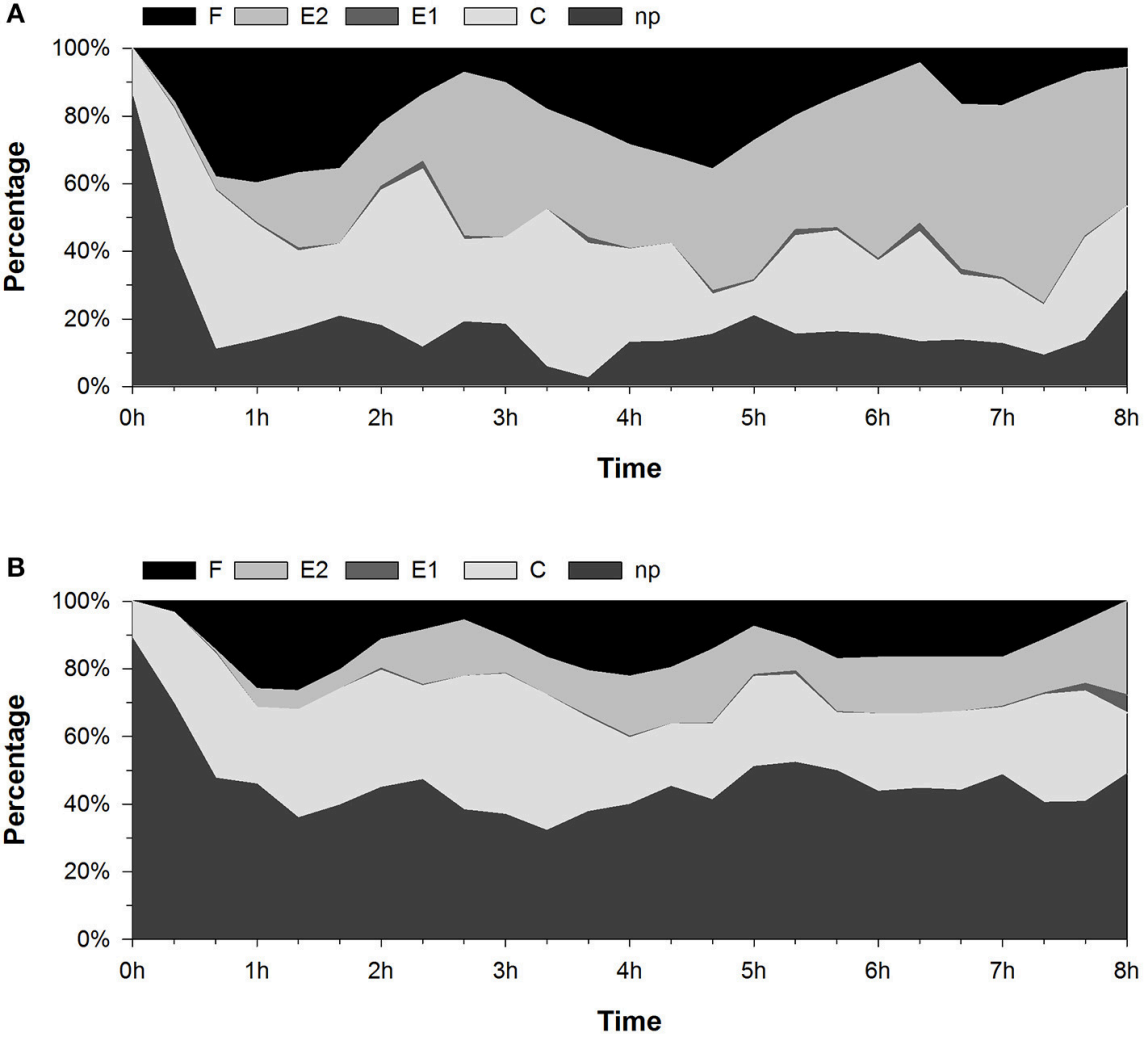
Percent change in the stylet activity of *Acyrthosiphon pisum* during 8-h EPG recordings. The data show the percentage of the duration of EPG waveforms in every 20-min interval. **(A)** LTA control group. **(B)** NPF treatment group.

The same behavior is illustrated in Figure 5, which shows the percentage of the duration of various EPG waveforms within 20-min intervals throughout the 8-h recording period. The percentage of the np waveform varied from 2.4 to 40.6% in the LTA control group and from 32.1 to 69.6% in the NPF group. The percentage of the E2 waveform varied from 1.7% to 63.7% in the LTA control group and from 0 to 27.8% in the NPF group. The delay in the occurrence of the C and E2 waveforms can also be observed in Figure 5. During the first hour, the maximum percentage of phloem ingestion reached 11.8% in the LTA control group, as shown by the E2 waveform, whereas the percentages of probing activity ranged from 34.3 to 47.0%. In contrast, the maximum percentage of phloem ingestion was only 5.6% in the NPF group, whereas the percentages of probing activity ranged from 22.7 to 36.9%.

### Influence of NPF silencing on pea aphid survival and reproduction

**Figure 6:**
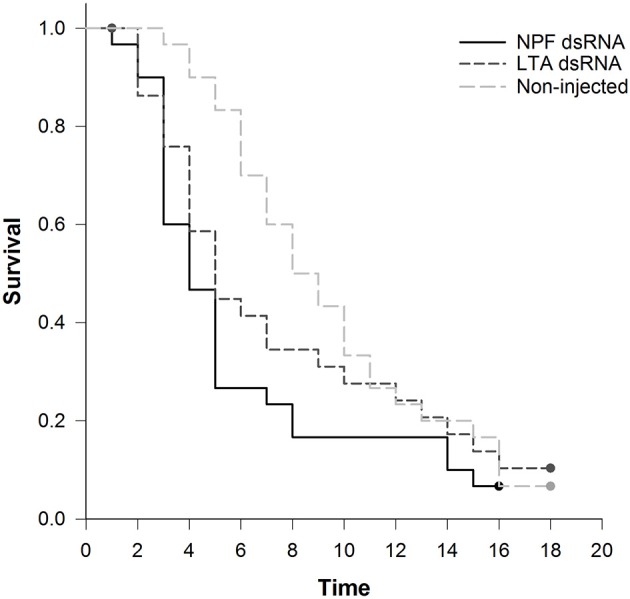
Influence of NPF gene silencing on *Acyrthosiphon pisum* survival. Kaplan-Meier survival log-rank analysis shows no significant difference in survival between the NPF treatment group and the two control groups (*P* > 0.05).

After microinjection of the NPF and LTA dsRNA, we monitored the aphids daily to evaluate their survival and reproduction until they no longer produced nymphs. The first dead aphid in the NPF treatment group was found 1 day after injection, whereas no mortality was observed during the first 48 h in the LTA control group (Figure 6). Kaplan-Meier survival log-rank analysis showed no significant difference in overall survival between the NPF treatment group and the two control groups, although the cumulative survival rate of the NPF group was lower than that of the control groups (χ^2^ = 2.040, df = 2, *P* > 0.05, Figure 6). This result indicated that NPF silencing does not affect aphid survival.

**Figure 7:**
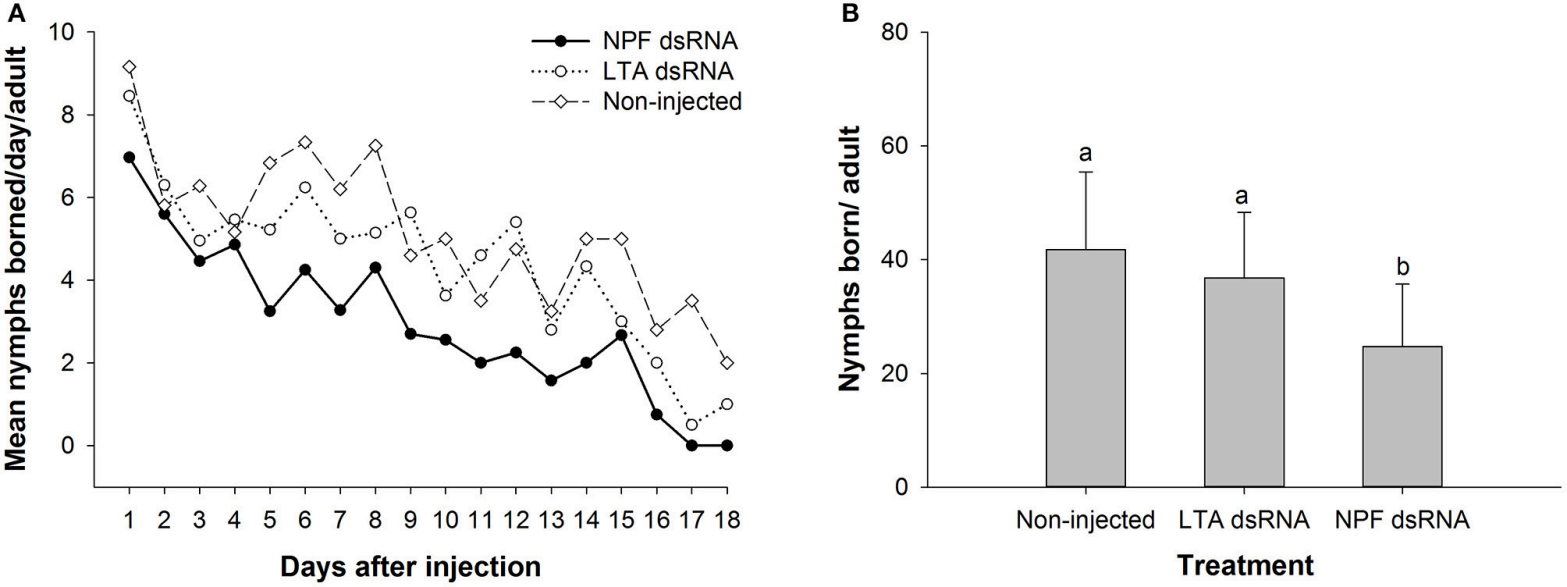
Influence of NPF gene silencing on *Acyrthosiphon pisum* reproduction. **(A)** Reproduction rates in the NPF group and the two control groups. The aphids in the NPF treatment group showed a lower reproduction rate during most of the observation period. **(B)** Total reproduction in the NPF treatment group was significantly lower than that in the control groups. The data were statistically analyzed by one-way ANOVA followed by Duncan's multiple-range test. Different lowercase letters indicate significant differences at the 0.05 level.

In both the NPF group and the two control groups, the reproduction rate reached its maximum level at the beginning of the observation period (Figure 7A). The maximum reproduction rates in the LTA group and non-injected group were ~ 8.5 and 9 nymphs per day, respectively, whereas the maximum rate in the NPF group was ~7 nymphs per day. From the 5th day on, the reproduction rate of the aphids in the NPF group obviously dropped compared with that of the two control groups (Figure 7A). The total number of offspring was significantly lower when the aphids were injected with NPF dsRNA, decreasing to 24.7 nymphs per adult, compared with 36.8 and 41.8 nymphs per adult in the LTA and the non-injected groups, respectively (*F* = 7.834, df = 2, 42, *P* < 0.05, Figure 7B).

## Discussion

### NPF and its receptor encoded in the pea aphid genome

This study extends the field of research on insect NPF signaling to aphids, where only one NPF-encoding gene was predicted (Huybrechts et al., 2010). This prediction is consistent with the results from the majority of insects studied to date, although there are some exceptions, such as the body louse and certain species of Lepidoptera, which exhibit two NPF-homologous genes (i.e., an NPY-encoding gene as well as an NPF-encoding gene Roller et al., 2008; Kirkness et al., 2010; Huang et al., 2011; Liu et al., 2013), and the NPF-encoding gene even produces two splicing variants (Roller et al., 2008; Liu et al., 2013). In this study, the *A. pisum* NPF and NPFR cDNA sequences were amplified via RT-PCR and re-sequencing. The results revealed that their corresponding protein sequences were identical to those predicted based on the sequence data from the *A. pisum* genome and EST database. The *A. pisum* NPF precursor consists of 158 amino acids. Similarly to other neuropeptide precursors, there is a signal peptide at the N-terminus, which putatively contains 23 amino acids and is responsible for translocation through the membrane of the endoplasmic reticulum (Huybrechts et al., 2010). In the prepropeptide of *A. pisum* NPF, the -RPRF sequence is followed by an amidation site (G) and a dibasic cleavage site (KR). Thus, after cleavage by the signal peptidase and carboxypeptidases and subsequent amidation by amidation monooxygenase, mature neuropeptide F is produced with an amidated RPRFamide C-terminus. This C-terminus is characteristic of the NPFs that have been identified in most insects and represents the ancestral form in invertebrates from an evolutionary perspective. In contrast, some hymenopteran species, such as *Apis mellifera* (Ament et al., 2011) and *Nasonia vitripennis* (Hauser et al., 2010), have an RYamide C-terminus, presumably derived from loss of the ancestral form of NPF after gene duplication (Nässel and Wegener, 2011). Although NPF belongs to one of the longest neuropeptide families, it appears that truncated NPF with only the eight to nine C-terminal amino acids can display biological activity (Van Wielendaele et al., 2013a,b; Sedra and Lange, 2016). An ortholog of the NPFR-encoding gene was also predicted in the pea aphid genome, and this gene produces four transcript splicing variants but exhibits a uniform open reading frame (ORF) with a length of 391 amino acids and the typical seven transmembrane domains.

### Transcriptional expression of NPF and NPFR in different developmental stages and body parts

qPCR is an efficient and widely used technique for monitoring gene expression at the mRNA level. In this study, it was employed to investigate the NPF and NPFR transcript levels at different ages and in different body parts of wingless pea aphids. Both the NPF and NPFR transcripts were shown to be present in different amounts in aphids of various ages, implying that the NPF signaling system might participate in the developmental regulation of some physiological processes or behaviors in aphids. It is worth noting that the low expression level of the receptor genes generally makes it difficult to detect any change in their expression by qRT-PCR, which might explain the lack of obvious changes in the NPFR expression levels among the different developmental stages, as shown in Figure 1A.

In terms of NPF localization, our results differed from those of previous studies. Information from other insect species studied to date has shown that NPF is mainly produced and secreted in the CNS and in the endocrine cells of the midgut (Brown et al., 1999; Stanek et al., 2002; Gonzalez and Orchard, 2008; Nuss et al., 2008, 2010; Huang et al., 2011; Van Wielendaele et al., 2013a), as indicated by its assigned name of the brain-gut peptide. Furthermore, in some insects, including *Helicoverpa assulta*, NPF mostly arises from endocrine cells in the midgut; i.e., the NPF expression level is higher in the midgut than in the brain (Liu et al., 2013). However, the midgut is not the only source of NPF in the insect digestive system; a previous RT-PCR analysis revealed the presence of an NPF transcript in the foregut of the termite *R. flavipes* (Nuss et al., 2010). Additionally, both NPF and NPY mRNA have been detected at lower levels in the foregut of 5th instar larvae of *H. assulta* (Liu et al., 2013). Although the presence of NPF in the midgut has been well demonstrated at the transcript or peptide level in various insect species, exceptions remain: NPF mRNA has not been found in the midgut endocrine cells of either 5th instar or adult *Rhodnius prolixus* but has been found in the hindgut (Gonzalez and Orchard, 2008; Sedra and Lange, 2016). Thus, it was surprising that NPF mRNA was not detected in the *A. pisum* intestine in the present study, indicating that endocrine cells in the aphid gut may not produce NPF mRNA or produce it only rarely. Furthermore, because the presence of mRNA is a prerequisite for production of a bioactive mature peptide, NPF might be absent in the aphid gut and might therefore not function in the aphid digestive system.

Although less research has been performed on the localization and distribution of NPF receptor mRNA than on NPF mRNA, the expression of the NPFR transcript in the aphid head and digestive system is consistent with the localization data obtained for other insect species (Garczynski et al., 2002; Nässel and Wegener, 2011; Deng et al., 2014). For instance, *in situ* hybridization experiments have revealed abundant NPFR expression in the brain, midgut, and accessory nerve of 3rd instar *Drosophila* (Garczynski et al., 2002). The NPFR-encoding transcript was also detectable in the aphid body remaining after removal of the head, gut and embryo, probably due to its extensive pattern of expression in insects. For example, the NPFR transcript has been observed in the testis, midgut, ovary, brain, fat body, epidermis, Malpighian tubule, and silk gland of *B. mori* larvae (Deng et al., 2014).

### Transcriptional expression levels of NPF and NPFR in different feeding states

We also demonstrated the effects of starvation and re-feeding on the expression of NPF and NPFR in aphids by comparing the transcript levels between starved and subsequently re-fed aphids and aphids that were fed in leaf cages/*ad libitum* on broad bean plants (Figure 2). The aphids in the different experimental groups included in this assay were collected simultaneously to exclude the possible influence of circadian rhythm on the NPF transcript expression levels. Despite quantitative differences among different time points, starvation resulted in up-regulation of the NPF transcript level, while re-feeding caused its down-regulation to the original level. This result was consistent with those previously reported in several other insect species. NPF expression is higher when these insects exhibit a desire for food or are feeding. In *D. melanogaster*, NPF expression is high in the brain of larvae attracted to food, and its down-regulation coincides with the display of behavioral phenotypes of older larvae, including hypermobility and feeding cessation (Wu et al., 2003). Similarly, in 5th instar *H. zea* larvae, the NPF levels in the hemolymph and midgut increase during the feeding and weight-gaining stages and then decrease with feeding cessation and the onset of metamorphosis, which is characterized by purging of the gut contents and wandering in search of a pupation site (Huang et al., 2011). The NPF transcript level has also been shown to be higher in starved desert locust, *Schistocerca gregaria*, than in those fed *ad libitum*, while the transcript level is significantly reduced in tissues such as the brain, optic lobes and midgut after a meal (Van Wielendaele et al., 2013a). However, the timing of the change in the NPF transcript levels differs between the desert locust and the pea aphid; the response of the NPF mRNA level to feeding and the nutritional state is more rapid in the former than in the latter, possibly because of the different feeding habits of these two insect species. It is also worth noting that no significant up- or down-regulation of the NPFR transcript level was observed in either starved or re-fed aphids (Figure 2B). These results indicate that the feeding state influences the expression of the NPF-encoding transcript in pea aphids, whereas its receptor might exhibit a more constant state under different physiological conditions. Taken together, these observations suggest a role for the neuropeptide F pathway in the response of wingless parthenogenetic pea aphids to their nutritional status, and this role involves regulation of the expression of NPF.

In this study, we monitored the changes in NPF/NPFR contents at the mRNA level by qRT-PCR in pea aphids at different developmental stages, in different body parts and under different physiological conditions. However, the contents of the neuropeptide and its mRNA do not necessarily correspond. For example, a high level of cholecystokinin mRNA in the pig cerebellum has been shown to not generate any detectable mature peptide (Gubler et al., 1987). Due to the complexity of neuropeptide production and excretion, particularly regarding post-translational modification and hydrolysis, the regulation observed at the mRNA level might not lead to a corresponding change in the production or release of the peptide.

### Effect of NPF on food intake and feeding behavior in aphids

We used RNAi technology to investigate the possible function of NPF in pea aphid feeding. RNAi has proven successful in the study of gene function but is difficult to achieve in the brain tissue of nematodes (Kennedy et al., 2004). In contrast, efficient interference in brain tissue has been reported in some lepidopteran species (Griebler et al., 2008; Hossain et al., 2008; Rodríguez-Cabrera et al., 2010). In *A. pisum*, NPF is mainly expressed in the head (at ~50- to 4000-fold higher levels than in other body parts, Figure 1B) and up to ~50% knockdown of NPF mRNA was detected in the whole aphid body after RNAi (Figure 3A). Thus, we can infer that RNAi is effective in the head of pea aphids, as reported by Wang et al. (2016).

Regulation of feeding was the first demonstrated function of NPF in *Drosophila* (Shen and Cai, 2001; Wu et al., 2003). Thereafter, NPF was shown to be involved in feeding or foraging in several other insect species (Gonzalez and Orchard, 2008; Huang et al., 2011; Van Wielendaele et al., 2013a), where it usually appears to play a positive role in stimulating food intake. For example, older *Drosophila* larvae stop feeding and display hypermobility to search for a pupation site. NPF overexpression in older larvae suppresses the above-mentioned behaviors, while loss of NPF signaling in young larvae leads to their premature display (Wu et al., 2003). In *S. gregaria*, NPF injection can increase food intake even when the animals display high NPF transcript levels at the time of hunger and show motivation for food (Van Wielendaele et al., 2013a).

Considering the stronger down-regulatory effect observed in the NPF-knockdown experiment than in the NPFR*-*knockdown experiment, we examined the change in food intake in pea aphids injected with NPF dsRNA. We found that the down-regulation of NPF expression significantly reduced food intake (Figure 4), which suggests that silencing of NPF expression in aphids inhibits their appetite, likely resulting in changes in feeding behavior and a consequent reduction of food intake, as observed in *S. gregaria* (Van Wielendaele et al., 2013a). Therefore, we subsequently analyzed the change in aphid feeding behavior following NPF knockdown using EPG technology. The timing of the appearance and the duration of the E waveform in EPG experiments generally reveal the preference of insects for phloem sap (Prado and Tjallingii, 1994). Thus, the delay of phloem activity recorded in aphids injected with NPF dsRNA indicated a reduced appetite, which was also demonstrated by postponement of the initiation of penetration and extension of the non-probing phase. Moreover, the apparent reduction in phloem ingestion could directly explain the reduction in food intake in NPF-silenced aphids shown in Figure 4. Above all, the lower appetite and shorter phloem ingestion period account for the reduction in food intake detected in NPF*-*silenced aphids. Although a link was found between the NPF levels and feeding behavior in pea aphids, the specific modification mechanism remained unclear. Nonetheless, considering the high level of NPF mRNA in the aphid brain and its possible absence in the gut, as well as the high level of NPFR mRNA in both the brain and the gut, we hypothesize that the aphid brain might be responsible for synthesizing and releasing the NPF signal that promotes food intake, while both the gut and the brain might be the main body compartments that respond to this signal.

It is also worth noting that the regulation of feeding behavior involves changes in the expression of not only NPF but also its receptor in both vertebrates and invertebrates (Wu et al., 2005b; Yahya et al., 2006; Lingo et al., 2007). Unfortunately, we did not observe a phenotypic change after NPFR knockdown due to the low gene silencing efficiency of NPFR dsRNA, although we attempted this experiment many times. Thus, whether and how NPFR is regulated to modify feeding behavior in aphids must be further investigated and verified. In addition, we focused on the wingless pea aphid in this study, however, aphids display wing dimorphism (winged or wingless) to adapt to environmental changes and winged and wingless aphids exhibit different characteristics in terms of morphology, physiology, and behavior (Braendle et al., 2006). The availability of nutrition is considered an important factor influencing the transformation from wingless to winged aphids (Müller et al., 2001). Therefore, investigating the relationship between NPF signaling and feeding behavior among winged pea aphids would be valuable.

### Effect of NPF silencing on aphid survival and reproduction

In both the NPF group and the two control groups, the reproduction rates reached their maximum level at the beginning of the observation period, which began on the 4th day after the aphids reached the adult stage. The silencing of NPF expression suppressed feeding in pea aphids, but we only observed a significant influence on aphid reproduction and no influence on survival. There might be two possible reasons for this result: (1) NPF silencing might last for only several days, as observed by Jaubert-Possamai et al. (2007), and the nutritional deficiency resulting from our experimental conditions might therefore not have been sufficient to cause aphid death; (2) the aphids sacrificed their reproductive ability to ensure survival in response to the reduced availability of nutrients (Will and Vilcinskas, 2015).

## Conclusion

In conclusion, the *A. pisum* NPF transcript was found to be present in the head but not in the gut, whereas NPFR transcript was present in both the gut and the head. NPF appears to be related to the regulation of aphid food intake and feeding behavior and further influences reproduction, but not survival. However, the role of this peptide in the regulation of aphid feeding at the molecular, cellular, and neural circuit levels remains unclear and requires further investigation.

## Author contributions

XL, M-JQ, YZ, and T-XL: designed the study; XL and J-WL: performed the experiments; XL and M-JQ analyzed the data; XL, YZ, M-JQ, and T-XL wrote the manuscript.

### Conflict of interest statement

The authors declare that the research was conducted in the absence of any commercial or financial relationships that could be construed as a potential conflict of interest.
